# Para-aortic irradiation for stage I testicular seminoma: results of a prospective study in 675 patients. A trial of the German testicular cancer study group (GTCSG)

**DOI:** 10.1038/sj.bjc.6601867

**Published:** 2004-05-11

**Authors:** J Classen, H Schmidberger, C Meisner, C Winkler, J Dunst, R Souchon, L Weissbach, V Budach, W Alberti, M Bamberg

**Affiliations:** 1 Department of Radiation Oncology, Universitätsklinikum, Hoppe-Seyler-Strasse 3, D-72076 Tübingen, Germany; 2 Department of Radiation Oncology, Universitätsklinikum, Robert-Koch-Str. 40, D-37075 Göttingen, Germany; 3 Institute for Medical Information Processing, Tübingen University, Westbahnhofstr. 3, D-72076 Tübingen, Germany; 4 Department of Radiation Oncology, Universitätsklinikum, Fetscherstr. 74, D-01307 Dresden, Germany; 5 Department of Radiation Oncology, Universitätsklinikum, Dryanderstr. 4-7, D-06110 Halle/Saale, Germany; 6 Department of Radiation Oncology, Allgemeines Krankenhaus, Grünstr. 35, D-58095 Hagen, Germany; 7 Department of Urology, Euromed Clinic, Europaallee 1, D-90763 Fürth, Germany; 8 Department of Radiation Oncol, Klinikum Charité, Schumannstr. 20/21, D-10117 Berlin, Germany; 9 Department of Radiation Oncology, Universitätsklinikum, Martinistr. 52, D-20246 Hamburg, Germany

**Keywords:** testicular seminoma, stage I, para-aortic radiotherapy, toxicity, secondary malignancies

## Abstract

A prospective nonrandomised trial was performed in order to evaluate tumour control and toxicity of low-dose adjuvant radiotherapy in stage I seminoma with treatment portals confined to the para-aortic lymph nodes. Between April 1991 and March 1994, 721 patients were enrolled for the trial by 48 centres in Germany. Patients with pure seminoma and no evidence of lymph node involvement or distant metastases received 26 Gy prophylactic limited para-aortic radiotherapy. Disease-free survival at 5 years was the primary end point. With a median follow-up of 61 months, 675 patients with follow-up investigations were evaluable for this analysis. Kaplan–Meier estimates of disease-free and disease-specific survival were 95.8% (95% CI: 94.2–97.4) and 99.6% (95% CI: 99.2–100%) at 5 years and 94.9% (95% CI: 92.5–97.4%) and 99.6% (95% CI: 99.2–100%) at 8 years, respectively. A total of 26 patients relapsed. All except two were salvaged from relapse. In all, 21 recurrences were located in infradiaphragmatic lymph nodes without any ‘in-field’ relapse. Nausea and diarrhoea grade 3 were observed in 4.0 and 1.0% of the patients, respectively. Grade 3 late effects have not been observed so far. The results of our trial lend further support to the concept of limited para-aortic irradiation as the recently defined new standard of radiotherapy in stage I seminoma. There is no obvious compromise in disease-specific or disease-free survival compared to more extensive hockey-stick portals, which were used as standard portals at the time this study was initiated.

The prognosis of clinical stage I testicular seminoma is favourable, with cure rates after orchiectomy and adjuvant radiotherapy of approximately 95% (Zagars and Babaian, 1987; Dosmann and Zagars, 1993; Vallis *et al*, 1995; Bauman *et al*, 1998). Traditionally, the target volume of radiotherapy comprises infradiaphragmatic para-aortic and ipsilateral iliac lymph nodes (‘hockey-stick’ portals). Para-aortic lymph nodes are the primary site of testicular lymphatic drainage as has been demonstrated by early lymphangiography (Busch *et al*, 1965) and surgical lymphadenectomy studies (Mason *et al*, 1991). Thus, limitation of the treatment portals of irradiation to the para-aortic lymphatics may be sufficient for safe control of retroperitoneal micrometastases in stage I seminoma. Omitting pelvic radiotherapy carries the potential benefit of reducing scattered radiation to the contralateral testis, thus minimising the risk of treatment-induced infertility (Jacobsen *et al*, 1997). Furthermore, limitation of the treatment portals may reduce gastrointestinal toxicity as well as the risk of secondary malignancies after treatment (Travis *et al*, 1997).

In 1986, Willich *et al* (1986) reported promising results for limited para-aortic radiotherapy in stage I seminoma. This report encouraged us to initiate a prospective multicentre trial in order to further evaluate the potential of small-volume para-aortic irradiation. The aims of our study were to quantify acute side effects as well as late toxicity of irradiation, and to identify the pattern of recurrences after treatment in order to optimise radiotherapy portal definition. Subsequent to the commencement of our study, several small pilot series showed recurrence rates and an overall survival (OS) comparable to the results obtained with conventional hockey-stick treatment (Read and Johnston, 1993; Niewald *et al*, 1995; Kiricuta *et al*, 1996; Logue *et al*, 1998; Sultanem *et al*, 1998). Finally, in 1999, a large randomised MRC trial was published demonstrating that confinement of the treatment portals to the para-aortic region did not adversely affect the overall relapse rate compared to hockey-stick portals (Fossa *et al*, 1999). This trial therefore established para-aortic radiotherapy as the new standard of irradiation for stage I seminoma. In 2001, yet another randomised MRC trial (TE18/19) demonstrated that reduction of the para-aortic radiation dose to 20 Gy was safe without an increase in relapse rates as compared to 30 Gy (Jones *et al*, 2001).

Interim results of the per protocol population of our trial have been reported previously (Bamberg *et al*, 1999) lending further evidence to the role of para-aortic radiotherapy as the new standard of radiotherapy in stage I seminoma. We now present an intention-to-treat analysis of the entire study population after a median time to follow-up of 61 months.

## PATIENTS AND METHODS

### Patient selection and staging process

Patients with pure testicular seminoma in clinical stage I disease according to the Royal Marsden classification system were eligible for the trial (no evidence of metastases). High inguinal ablation of the tumorous testicle was required in every patient. The staging procedure comprised a computed tomography (CT) of abdomen and pelvis, a chest CT or chest X-ray, and analysis of tumour markers alpha-fetoprotein (AFP) and human chorionic gonadotropin (*β*-HCG) prior and subsequent to ablation of the testis. During the first year of the study, only patients with negative presurgical levels of *β*-HCG were admitted. During the second and third year of the trial, patients with elevated *β*-HCG levels of up to 200 IU l^−1^ were admitted as well, once there was evidence that initial *β*–HCG elevation did not adversely affect the prognosis of the patients (Mirimanoff *et al*, 1993). Exclusion criteria were a positive AFP level prior to orchiectomy, a history of prior abdominal/pelvic radiotherapy or chemotherapy, withdrawal of informed consent, concurrent severe diseases, or treatment with cobalt-60 machines. All patients were asked for informed consent according to the Declaration of Helsinki.

### Radiotherapy

Margins of the treatment portals were defined according to the following criteria. The upper field border was set to the cranial rim of the 11th thoracic vertebra, and the lower field margin was defined by the inferior border of the fourth lumbar vertebra. Lateral field margins were defined by the ends of the lateral vertebral processes, resulting in a width of the fields between 9 and 11 cm. Radiation portals were assigned using treatment simulators in all patients.

Radiotherapy was applied through ventro-dorsal opposing fields with 4–20 MV photons of linear accelerators. Both opposing fields were treated daily for five times a week with a fraction of 2.0 Gy day^−1^ as specified in the ICRU 29 report for opposing fields. A total dose of 26 Gy was applied in 17 days. If treatment interruptions of more than 3 days occurred, the total dose was increased to 30 Gy.

### Protocol violations

Protocol violations were classified as *major violations* (MAV) if they had either a potentially adverse effect on the therapeutic efficacy of radiotherapy, or if they were apt to increase treatment-related side effects (no chest imaging or no CT abdomen/pelvis for staging, no AFP or elevated AFP prior to ablation, incorrect stage assignment, dose prescription of less than 25 Gy or more than 34 Gy). Protocol violations were classified as *minor violations* (MIV) if no negative effect on treatment outcome or toxicity of irradiation was assumed.

### Follow-up

Follow-up examinations were performed every 3 months for the first 2 years after radiotherapy and every 6 months thereafter. Clinical examination, analysis of AFP and *β-*HCG, chest X-ray, and assessment of late toxicities were required at each visit. Computed tomography scans of abdomen and pelvis were taken twice a year for the first 2 years, and annually thereafter. Abdominal ultrasound was performed in turn with abdomino-pelvic CT scans (twice a year during the first 2 years, once a year after the second year).

### End points

The primary end point of the study was relapse-free survival at 5 years. Since a potent salvage chemotherapy is available for relapsing patients, disease-specific survival (DSS) was chosen as a secondary end point with an expected survival of at least 95% at 5 years. Furthermore, acute and late gastrointestinal and cutaneous toxicities (see below) were defined as secondary end points.

### Monitoring of side effects

Acute and late side effects of treatment (gastrointestinal and cutaneous/soft tissue effects) were recorded during radiotherapy and at each follow-up visit using the EORTC/RTOG scores.

### Data monitoring and data processing

The pathohistologic, diagnostic, therapeutic, and follow-up data were recorded on specially prepared forms and entered into a computerised database at the coordinating centre (Tübingen University) using the study monitoring system of the Institute for Medical Information Processing (IMI, Tübingen University). After closing of the database for this analysis (31 December 2001), all data were transferred to the IMI for further data processing.

The trial was designed as an observational study over a period of 3 years. With an expected population of 600 at 3 years, a one-sided 95% confidence interval for a single proportion using the large sample normal approximation will extend 1.3% from an expected proportion of 3.7%. Thus, a 3.7% crude relapse rate for the entire study population with a one-sided 95% confidence limit extending to 5% would ensure that a rate of 5% – considered to be the highest acceptable relapse rate – would not be surpassed. Failure from treatment was continuously monitored over the treatment period and early termination of the study was planned once the critical relapse rate was observed during the treatment period.

Continuous variables were described by use of statistical characteristics (means, standard deviations). Discrete variables are described as counts and percentages. Kaplan–Meier estimates and their 95% confidence intervals were computed for disease-free survival (DFS), OS, and DSS at 5 and 8 years after the end of radiotherapy. For statistical analysis, the database was converted into SAS files and the SAS system (SAS 6.11 for Windows) was used.

## RESULTS

Between April 1991 and March 1994, 721 patients were enrolled for the study by 48 institutions (see Appendix A). There was no follow-up information available in 43 patients. These patients were excluded from the analysis as were another three patients once informed consent had been withdrawn. Median time to follow-up of the remaining study population of 675 patients was 61 months (range 0.6–121 months). Median age of the patients was 34 years (range 16–73). A right-sided tumour was observed in 51.9% of the patients, 47.7% had a left-sided tumour, and three patients (0.4%) presented with bilateral seminoma. The distribution by tumour histology and T stage is shown in Table 1

**Table 1:** Distribution of primary tumours by histology and T stage according to 1987 TNM classification of UICC

**Histology**	**Patients *n* (%)**
Classical seminoma	641 (95)
Spermatocytic seminoma	10 (1.5)
Anaplastic seminoma	19 (2.8)
No further subclassification	5 (0.7)
	
**T stage**	**Patients n (%)**
1	565 (83.7)
2	94 (13.9)
3	14 (2.1)
4	0 (0)
Unknown	2 (0.30)

. In all, 82 patients (12.2%) presented with an elevated *β*-HCG prior to ablation testis.

### Protocol violations

A total of 485 patients (71.9%) were staged and treated strictly per protocol (PP), while 115 (17%) and 75 patients (11.1%) had major or minor protocol violations, respectively. Median radiation dose for PP, MAV, and MIV was 26 Gy (range 25–34 Gy).

### Tumour control and survival

In all, 26 patients have relapsed from treatment (18 PP, four MAV, and four MIV). Of 26 recurrences, 22 were located in infradiaphragmatic lymph nodes. There was one mediastinal and one supraclavicular relapse. Three patients developed distant metastases. A total of 24 patients were salvaged by chemotherapy or irradiation (Table 2

**Table 2:** Recurrence from testicular cancer after adjuvant radiotherapy

**No.**	**Primary tumour location**	**Time to relapse**	**Location of recurrent disease**	**Histology of relapse**	**Treatment for relapse**	**Status**	**Second relapse**
1	Left	9	Liver, lung, iliac left	Seminoma	4 × PEI+IF-RT left iliac 36 Gy	CR	No
2	Left	25	Th 8–10 with intraspinal tumour growth	ND	IF-RT 40.5 Gy+2 × PEB	CR	No
3	Right	11	C4–6 and cervical lymph nodes	ND	4 × PEB+IF-RT 30 Gy	CR	No
4	Right	89	Vesicular seminalis right^a^	Seminoma	4 × carboplatin, on local relapse in vesicula seminalis: pelvic RT to 26 Gy and prostatic boost to 30 Gy	CR	No
5	Left	28	Left supraclavicular^b^	Seminoma and lymphoma	Mantle-field RT 30 Gy	CR	No
6	Right	27	Mediastinal	Seminoma	6 × PEB	CR	No
7	Left	26	Left iliac+mediastinal	ND	3 × PEB+surgery	CR	No
8	Left	3	Left kidney hilum	Seminoma	Surgery	Dead	No
9	Left	28	Left iliac+left kidney hilum	ND	4 × PEB, 2 × PEI, surgery for residual lymphoma	CR	No
10	Left	13	Right upper iliac commune, bifurcation	Seminoma	Surgery+3 × PEB	CR	No
11	Left	47	Low para-aortic iliac left	Seminoma	Surgery+3 × PEB	CR	No
12	Left	12	Left kidney hilum and iliac commune left	Not known	4 × PEB	CR	No
13	Right	19	Right inguinal	Seminoma	Surgery+IF-RT 26 Gy	CR	No
14	Left	4	Left inguinal	Seminoma	IF-RT 30 Gy	CR	No
15	Right	9	Iliac right, bifurcation	ND	4 × PEB	CR	No
16	Right	6	Right iliac	Not known	3 × PEI	CR	No
17	Left	6	Left iliac	Not known	3 × PEB	CR	No
18	Left	39	Left inguinal^c^	Seminoma	Surgery+3 × PEV	CR	No
19	Left	15	Bilateral inguinal/iliac	Seminoma	Surgery (inguinal left)+RT bilateral iliac/inguinal and left scrotal: 42 Gy	CR	34 months after PD: para-aortic/iliac and mediastinal; CR after 4 × CEB
20	Right	21	High iliac right and para-aortic right	ND	Refused salvage treatment	Dead	No
21	Right	1	Right kidney hilum	ND	2 × PEB	CR	No
22	Left	13	Iliac commune left/external iliac	Seminoma	Surgery+chemotherapy, schedule unknown	CR	No
23	Left	11	Left inguinal/iliac	Seminoma	Surgery+IF-RT 36 Gy	CR	40 months after PD: inguinal left; CR after surgery+2 × PEB
24	Left	16	Iliac left/inguinal	Seminoma	4 × PEB+1 × PEI	CR	No
25	Left	1	Iliac right	ND	3 × PEB	CR	No
26	Left	35	Left kidney hilum, para-aortic	ND	4 × PEI	CR	No

ND=not done; CR=complete remission; PEB= cisplatin, etoposide, bleomycin; PEI= cisplatin, etoposide, ifosfamide; PEV=cisplatin, etoposide, vincristin; CEB=carboplatin, etoposide, bleomycin; IF-RT=involved-field radiotherapy; RT=radiotherapy; PD=primary diagnosis.

a Patient suffered from second tumour (meningioma).

b Recurrence combined with cc-cb-lymphoma.

c Previous herniotomy.

, Figure 1

**Figure 1:**
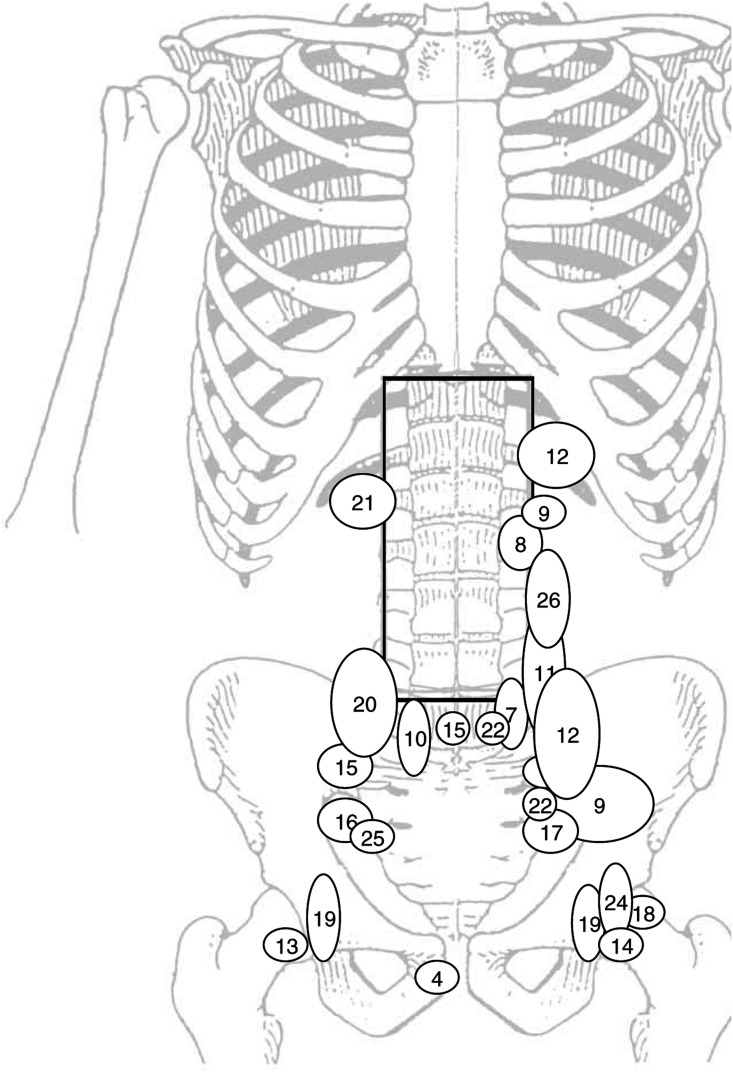
Locations of infradiaphragmatic recurrences. The numbers refer to Table 2.

). There was no ‘in-field’ relapse except for one patient who on review of the initial CT scans was found to have stage IIB seminoma. This patient progressed rapidly after radiotherapy, was submitted to lymphadenectomy, and died from cerebral embolism after surgery. A second patient suffering from retroperitoneal recurrence refused salvage chemotherapy and died of progressive disease. This patient was part of the PP population. In addition, there were four nonseminoma-related deaths. On relapse, 11 patients (1.63%) had tumour involving the ipsilateral pelvis. However, isolated ipsilateral recurrence was rare with only four patients affected (0.59%). Median time to relapse was 14 months (range 0–86 months). Disease-free survival for PP, MAV, and MIV were 96.1% (95% CI: 94.2–97.9%), 95.8% (95% CI: 91.7–99.9%), and 94.1% (95% CI: 88.3–99.7%) at 5 years, and 94.9% (95% CI: 92.0–97.8%), 95.8% (95% CI: 91.7–99.9%), and 94.1% (95% CI: 88.3–99.7%) at 8 years, respectively. Disease-free survival and DSS for the entire population were 95.8 (95% CI: 94.2–97.4%) and 99.6% (95% CI: 99.2–100%) at 5 years, and 94.9% (95% CI: 92.5–97.3%) and 99.6% (95% CI: 99.2–100%) at 8 years, respectively (Figure 2

**Figure 2:**
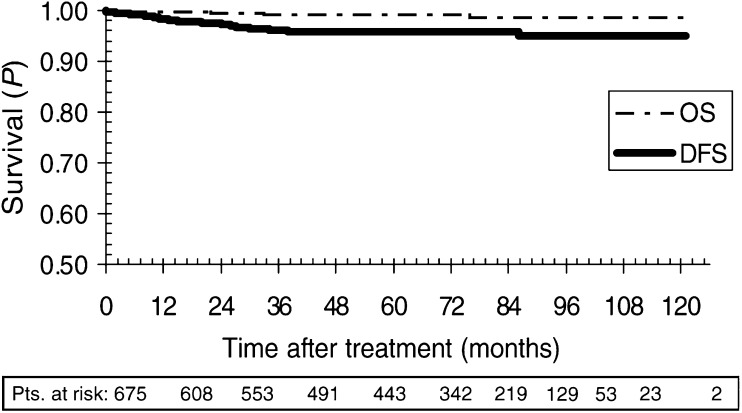
Kaplan–Meier curve for the entire study population. Pts=patients; DFS=disease-free survival, OS=overall survival.

). There was no statistically significant difference between the three study populations for DFS or DSS (log rank *P*=0.71). Overall survival at 5 and 8 years was 99.1% (95% CI: 98.4–99.9%) and 98.6% (95% CI: 97.3–99.9%), respectively.

### Acute and late toxicity

Maximum acute toxicity of radiotherapy was dominated by nausea grade I, which was observed in 46.1% of all patients (Table 3

**Table 3:** Maximum acute toxicity of radiotherapy assessed for skin, nausea, and diarrhoea

**EORTC/RTOG grade**	**Skin**	**Nausea**	**Diarrhoea**
0	94.3	42.8	88.0
1	5.0	46.1	9.6
2	0.3	6.7	1.0
3	0	4.0	1.0
4	0	0	0
Unknown	0.4	0.4	0.4

). Grade 2 and 3 nausea was documented in 6.7 and 4.0% of the patients, respectively. Skin toxicity was mild with grade 1, 2, and 3 side effects in 5.0, 0.3, and 0%, respectively. Likewise, diarrhoea was infrequent with grade 1, 2, and 3 toxicity in 9.6, 1.0, and 1.0% of the patients. There were no grade 4 side effects. Furthermore, there were no statistically significant differences in acute toxicity between the PP, MAV, or MIV populations.

On follow-up, four patients (0.6%) exhibited slight hyperpigmentation in the former treatment field (grade 1), and one patient (0.2%) developed telangiectasia (grade 2). A mild subcutaneous fibrosis EORTC (grade 1) was documented in one patient (0.2%). In all, 10 patients (1.5%) reported occasional diarrhoea (grade 1) and one patient (0.2%) had occasional diarrhoea with slight cramps (grade 2). No grade 3 or 4 late toxicity has been observed.

### Secondary tumours

Secondary tumours were observed in 17 patients (2.5%). Among these, there were seven contralateral testicular cancers and one contralateral carcinoma *in situ*. Nontesticular tumours comprised centroblastic–centrocytic lymphoma, acute leukaemia, meningioma, glioblastoma, head-and-neck cancer, gastrointestinal cancers, and nasal basalioma. Four patients died due to uncontrolled secondary malignancies.

## DISCUSSION

Adjuvant radiotherapy has been the standard treatment for stage I seminoma for decades. However, optimal management of the patients is still a matter of controversy (Milosevic *et al*, 1999; Classen *et al*, 2001). In spite of high cure rates achieved with adjuvant radiotherapy, efforts have been made to introduce alternative treatment strategies that would potentially reduce major side effects of irradiation. Among these, impairment of fertility by scattered radiation to the contralateral testis (Jacobsen *et al*, 1997), gastrointestinal morbidity (Fossa *et al*, 1989), and the risk of radiation-induced malignancies (Travis *et al*, 1997) are of major concern. In order to avoid these side effects, a policy of ‘watch and wait’ has been evaluated at some centres (Maase *et al*, 1993; Warde *et al*, 1997) applying treatment only to those patients suffering from relapse. Other study groups have investigated the role of single-agent carboplatin as adjuvant treatment (Oliver *et al*, 1994; Dieckmann *et al*, 2000).

Yet another strategy to limit side effects of adjuvant treatment is to minimise the target volume of radiotherapy by confining the treatment portals to the para-aortic lymph nodes, which have previously been shown to be the site of primary lymphatic drainage of the testicles (Busch *et al*, 1965). Our trial reported here is the largest prospective study evaluating the role of limited para-aortic radiotherapy in stage I seminoma in a nonrandomised setting. The low relapse rate observed in our study compares favourably to results of previously published pilot series using this treatment schedule (Willich *et al*, 1986; Niewald *et al*, 1995; Kiricuta *et al*, 1996; Logue *et al*, 1998; Sultanem *et al*, 1998). While these reports were limited by mostly retrospective analysis of the patients and small study populations, our trial now provides profound evidence for the feasibility and safety of limited para-aortic radiotherapy in stage I seminoma in a sufficiently large number of patients.

There was no follow-up information available in 43 patients who were consequently excluded from the analysis. Owing to the small proportion of patients excluded, it is unlikely that this would have had a relevant influence on the calculation of survival parameters and the principle conclusion of our trial that para-aortic radiotherapy yields excellent results with respect to tumour control.

The rate of ipsilateral iliac relapses observed in our patients was low (1.63%). This rate is well in the range of 0–2.2% observed by other authors for limited para-aortic treatment (Kiricuta *et al*, 1996; Logue *et al*, 1998; Sultanem *et al*, 1998). It can, however, be speculated that inclusion of ipsilateral iliac lymph nodes into the target volume might have prevented relapse from seminoma in some of our patients. Yet, at the same time, routine treatment of the pelvis would have been of no value in more than 98% of the patients merely contributing to treatment-related toxicity instead. This finding of only a very small benefit from pelvic irradiation is supported by a randomised MRC trial directly comparing para-aortic treatment to conventional hockey-stick radiotherapy (Fossa *et al*, 1999). The trial reported a significant but very small increase in pelvic recurrences of 1.8% when omitting iliac treatment.

Any increase in the pelvic recurrence rate after para-aortic treatment has to be weighted against the overall DFS in these patients. When comparing the relapse rate of 5.1% observed in our trial (including one patient with initial stage IIB seminoma) to reported recurrence rates of 2–6% for conventional ‘hockey-stick’ irradiation (Zagars and Babaian, 1987; Vallis *et al*, 1995; Bauman *et al*, 1998), there seems to be no obvious compromise in overall tumour control by omitting pelvic radiotherapy. Finally, with a disease-specific mortality of less than 1%, survival was not compromised in our study. These findings are again supported by the previously mentioned MRC trial, which could reliably exclude an increase in the overall relapse rate of more than 4.6% and a decrease in survival of more than 1.7% for patients treated with limited para-aortic radiotherapy (Fossa *et al*, 1999).

Our study is limited by the nonrandomised trial design, and conclusions drawn from this study might be considered to be less compelling. However, it has recently been demonstrated that well-designed observational trials do not systematically overestimate the magnitude of treatment effects as compared to randomised studies (Benson and Hartz, 2000; Concato *et al*, 2000). Therefore, with a large number of homogeneously managed patients, our study provides sound evidence that omission of pelvic treatment is safe without a clinically relevant increase in the overall relapse rate.

Protocol violations observed in 28% of our patients are of major concern, since nonadherence to protocol requirements might impact on treatment outcome. However, neither DFS nor acute toxicity was significantly influenced by conservatively defined major or minor protocol violations, respectively. This finding indicates that protocol violations documented in our trial do in fact not confound our results. They rather reflect some heterogeneity in treatment quality, which may be expected in any trial with a large number of participating centres.

With four patients recurring with involvement of the ipsilateral kidney hilum and another three patients failing near the lower field border at high iliac commune lymph nodes, moderate extension of the portals to cover these areas might have prevented relapse from seminoma in some of our patients. In fact, field alignment in the recent MRC trials (Fossa *et al*, 1999; Jones *et al*, 2001) included the kidney hilum ipsilateral to the primary tumour and covered one more lumbar vertebra including L5. This moderate extension of the portals is not expected to increase significantly toxicity of radiotherapy but rather bears the potential to further lower locoregional relapse rates of irradiation. Based on the result from our trial, we recommend to extend standard field margins to cover the fifth lumbar vertebra and to include the ipsilateral kidney hilum.

Computed tomography scans of abdomen and pelvis for follow-up were mandatory in our trial. However, the majority of patients with infradiaphragmatic recurrence had relapse that involved the pelvis. Therefore, the need for abdominal CT scans is questionable since the detection rate of isolated abdominal recurrences was in fact very low.

The radiation dose of 26 Gy applied in our trial, although somewhat lower than 30–35 Gy recommended by other European authors (Fossa *et al*, 1989, 1999; Kiricuta *et al*, 1996), is sufficient for control of microscopic seminoma, since no true in-field recurrence was observed. Furthermore, there is now convincing evidence that a further dose reduction to 20 Gy is safe without compromise in tumour control as has recently been demonstrated by MRC trials TE18/19 (Jones *et al*, 2001).

We observed a strikingly low incidence of acute toxicity. In addition, no major late toxicity like duodenal ulcers, or gastrointestinal discomfort, which have been observed by others (Fossa *et al*, 1989), was noted in our patients. Fossa *et al* (1999) reported a considerably higher rate of acute grade 2 and 3 side effects of 14 and 11% nausea, respectively. The radiation dose used in their trial was 30 Gy as compared to 26 Gy in our study. A further dose reduction to 20 Gy can therefore be expected to translate into an even more favourable profile of toxicity (Jones *et al*, 2001) thus beneficially impacting on the quality of life of the patients. Furthermore, the shortened treatment schedule will possibly reduce the days off work. These benefits of the reduced treatment dose may ultimately lower the socioeconomic costs caused by stage I seminoma. Finally, reducing the radiation dose to 20 Gy may also be beneficial with respect to the risk of radiation-induced secondary malignancies. These are a serious concern in seminoma patients, and a significant risk of radiation-induced tumours has previously been demonstrated (Travis *et al*, 1997). A total of 17 patients in our series suffered from secondary tumours, and nine of these were nontesticular events. Considering this low rate of nontestis tumour events, there is no obvious excess of secondary malignancies, but longer follow-up is warranted for a more reliable assessment of the risk of secondary cancers in this cohort of seminoma patients as the reported latency is some 10–15 years (Travis *et al*, 1997).

To overcome potential disadvantages of adjuvant radiotherapy, single-agent carboplatin chemotherapy has gained increasing interest in recent years. Several small pilot studies demonstrated low relapse rates in the range of 3–5% using 1–2 courses of carboplatin (Oliver and Ong, 1996; Krege *et al*, 1997; Dieckmann *et al*, 2000). The potential advantage of this treatment option may be the reduced treatment time, treatment of micrometastasis outside the strictly defined portals of radiotherapy, reduction in the risk of secondary malignancies, and improvements in treatment-related toxicities. However, equivalence of adjuvant radiotherapy and carboplatin chemotherapy in terms of relapse rates has yet to be demonstrated, and two randomised trials are currently conducted by the MRC and the German Testicular Cancer Study Group (GTCSG) to clarify this question. Yet another potential alternative to adjuvant radiotherapy is the surveillance strategy. The intention of this approach is to reserve active treatment to those patients relapsing after primary orchidectomy and to spare the majority of patients any potentially toxic adjuvant treatment. Several study groups could demonstrate that the DSS of the patients managed by surveillance is not compromised as compared to radiotherapy. The rate of relapse is in the range of 14–19%, and the DSS approaches 100% (Horwich *et al*, 1992; Maase *et al*, 1993; Oliver *et al*, 1994; Warde *et al*, 1997). In a pooled analysis of the three largest surveillance protocols, the tumour size (>4 cm) and tumour invasion of the rete testis were identified as independent prognostic factors for relapse (Warde *et al*, 2002). These risk factors may help to identify patients with a high or low risk of relapse and may be beneficial for information of the patient in the process of decision-making in adjuvant treatment. However, a risk-adapted surveillance strategy using these factors has up to date not prospectively been evaluated.

In conclusion, our trial provides further evidence in a large study population with long-term follow-up that limited para-aortic irradiation in stage I testicular seminoma is safe and feasible yielding excellent cure rates at a very low rate of treatment-related toxicity. In comparison to published data on conventional hockey-stick treatment, the rate of recurrences is not increased. Those patients suffering from relapse can be cured by systemic treatment or radiotherapy. Judging from our trial and the previously reported randomised study (Fossa *et al*, 1999), we consider limited para-aortic treatment for stage I seminoma as the new standard of radiotherapy (Krege *et al*, 2001) against which other potential alternatives like surveillance or adjuvant carboplatin chemotherapy have to be compared. We recommend standard field margins extending from Th11 to the fifth lumbar vertebra and including the ipsilateral kidney hilum.

## Appendix A

### LIST OF PARTICIPATING CENTRES IN GERMANY

XRT Stadt- und Kreiskrankenhaus, Ansbach; XRT Klinikum, Aschaffenburg; XRT Krankenhauszweckverband, Augsburg; XRT Klinikum, Bamberg; XRT Klinikum, Bayreuth; XRT Charité, Berlin; URO Klinikum am Urban, Berlin; XRT Klinikum Steglitz, Berlin; XRT Johannes-Krankenhaus, Bielefeld; XRT St Agnes-Hospital, Bocholt; XRT Städt. Kliniken, Darmstadt; XRT Universitätsklinikum, Dresden; XRT Klinikum, Duisburg; XRT Universitätsklinikum, Düsseldorf; XRT Universitätsklinikum, Erlangen; URO Marienhospital, Erwitte; XRT Universitätsklinikum, Essen; XRT Krupp-Krankenhaus, Essen; XRT Krankenhaus Nordwest, Frankfurt; XRT Universitätsklinikum, Freiburg; URO Klinikum, Fulda; XRT Krankenhaus am Eichert, Göppingen; XRT Universitätsklinikum, Göttingen; XRT Städtisches Krankenhaus, Gütersloh; XRT Allgemeines Krankenhaus, Hagen; XRT Universitätsklinikum, Halle; URO Bundeswehrkrankenhaus, Hamburg; XRT Universitätsklinikum, Eppendorf, Hamburg; XRT Marienhospital, Hamm; XRT Universitätsklinikum, Heidelberg; XRT Städtische Krankenanstalt, Heilbronn; XRT Städtische Klinik, Karlsruhe; XRT St Vincentius Krankenhaus, Karlsruhe; XRT Universitätsklinikum, Köln; XRT Klinikum, Konstanz; XRT Städt. Krankenanstalten, Krefeld; XRT Universitätsklinikum, Lübeck; XRT Klinikum, Ludwigsburg; URO Klinikum, Mannheim; XRT Evangelisches Krankenhaus, Mülheim/Ruhr; XRT Klinikum Nord, Nürnberg; XRT Kreiskrankenhaus, Offenburg; XRT Städt. Krankenhaus, Passau; XRT Winterberg-Krankenhaus, Saarbrücken; XRT Diakoniekrankenhaus, Schwäbisch-Hall; XRT Klinikum, Schwerin; XRT Katharinen Hospital, Stuttgart; XRT Mutterhaus der Borromäerinnen, Trier; XRT Universitätsklinikum, Tübingen.

XRT=Department of Radiation Oncology; URO=Department of Urology.

## References

[bib1] Bamberg M, Schmidberger H, Meisner C, Classen J, Souchon R, Weinknecht S, Schorcht J, Walter F, Engenhart-Cabillic R, Schulz U, Born H, Flink M (1999) Radiotherapy for stage I, IIA/B testicular seminoma. Int J Cancer 83: 823–827 10597202 10.1002/(sici)1097-0215(19991210)83:6<823::aid-ijc22>3.0.co;2-v

[bib2] Bauman GS, Venkatesan VM, Ago CT, Radwan JS, Dar AR, Winquist EW (1998) Postoperative radiotherapy for Stage I/II seminoma: results for 212 patients. Int J Radiat Oncol Biol Phys 42: 313–317 9788409 10.1016/s0360-3016(98)00227-2

[bib3] Benson K, Hartz AJ (2000) A comparison of observational studies and randomized, controlled trials. N Engl J Med 342: 1878–1886 10861324 10.1056/NEJM200006223422506

[bib4] Busch FM, Sayegh ES, Chenault OW (1965) Some uses of lymphangiography in the management of testicular tumors. J Urol 93: 490–495 14276208 10.1016/S0022-5347(17)63811-4

[bib5] Classen J, Souchon R, Hehr T, Bamberg M (2001) Treatment of early stage testicular seminoma. J Cancer Res Clin Oncol 127: 475–481 11501746 10.1007/s004320100243 PMC12164820

[bib6] Concato J, Shah N, Horwitz RI (2000) Randomized, controlled trials, observational studies, and the hierachy of research designs. N Engl J Med 342: 1887–1892 10861325 10.1056/NEJM200006223422507 PMC1557642

[bib7] Dieckmann K-P, Brüggeboes B, Pichlmeier U, Küster J, Müllerleile U, Bartels H (2000) Adjuvant treatment of clinical stage I seminoma: is one single course of carboplatin sufficient? Urology 55: 102–106 10654903 10.1016/s0090-4295(99)00376-3

[bib8] Dosmann MA, Zagars GK (1993) Postorchiectomy radiotherapy for stages I and II testicular seminoma. Int J Radiat Oncol Biol Phys 26: 381–390 7685748 10.1016/0360-3016(93)90954-t

[bib10] Fossa SD, Aass N, Kaalhus O (1989) Long-term morbidity after infradiaphragmatic radiotherapy in young men with testicular cancer. Cancer 64: 404–408 2736486 10.1002/1097-0142(19890715)64:2<404::aid-cncr2820640211>3.0.co;2-7

[bib9] Fossa SD, Horwich A, Russell JM, Roberts JT, Cullen MH, Hodsen NJ, Jones WG, Yosef H, Duchesne GM, Owen JR, Grosch EJ, Chetiyawardana AD, Reed NS, Widmer B, Stenning SP (1999) Optimal planning target volume for stage I testicular seminoma: a Medical Research Council randomized trial. J Clin Oncol 17: 1146–1154 10561173 10.1200/JCO.1999.17.4.1146

[bib11] Horwich A, Alsanjari N, Hern RA, Nicholls J, Dearnaley DP, Fisher C (1992) Surveillance following orchiectomy for stage I testicular seminoma. Br J Cancer 65: 775–778 1586607 10.1038/bjc.1992.164 PMC1977398

[bib12] Jacobsen KD, Olsen DR, Fossa K, Fossa SD (1997) External beam radiotherapy in patients with seminoma stage I: field type, testicular dose, and spermatogenesis. Int J Radiat Oncol Biol Phys 38: 95–102 9212009 10.1016/s0360-3016(96)00597-4

[bib13] Jones WG, Fossa SD, Mead GM, Roberts JT, Sokal M, Naylor S, Stenning SP (2001) A randomised trial of two radiotherapy schedules in the adjuvant treatment of stage I seminoma (MRC TE 18). Eur. J. Cancer 37(Suppl 6): 157 (abstract)

[bib14] Kiricuta IC, Sauer J, Bohndorf W (1996) Omission of pelvic irradiation in stage I testicular seminoma: a study of postorchiectomy paraaortic radiotherapy. Int J Radiat Oncol Biol Phys 35: 293–298 8635936 10.1016/0360-3016(96)00093-4

[bib16] Krege S, Kalund G, Otto T, Goepel M, Rübben H (1997) Phase II study: adjuvant single-agent carboplatin therapy for clinical stage I seminoma. Eur Urol 31: 405–407 9187898 10.1159/000474497

[bib15] Krege S, Schmoll HJ, Souchon R (2001) Interdisciplinary consensus on diagnosis and treatment of testicular germ cell tumors: results of an update conference on evidence-based medicine (EBM). Eur Urol 40: 372–391 11713391 10.1159/000049804

[bib17] Logue JP, Mobarek N, Read G (1998) Short fractionation para-aortic radiotherapy for stage I seminoma of the testis. Radiother Oncol (48Suppl 1): S10 (abstract)

[bib18] Maase H von der, Specht L, Jacobsen GK, Jakobsen A, Madsen EL, Pedersen M, Rorth M, Schultz H (1993) Surveillance following orchiectomy for stage I seminoma of the testis. Eur J Cancer 29A: 1931–1934 8280484 10.1016/0959-8049(93)90446-m

[bib19] Mason MD, Featherstone T, Olliff J, Horwich A (1991) Inguinal and iliac lymph node involvement in germ cell tumours of the testis: implications for radiological investigation and for therapy. Clin Oncol 3: 147–150 10.1016/s0936-6555(05)80835-0 1676904

[bib20] Milosevic M, Gospodarowicz M, Warde P (1999) Management of testicular seminoma. Semin Surg Oncol 17: 240–249 10588852 10.1002/(sici)1098-2388(199912)17:4<240::aid-ssu4>3.0.co;2-q

[bib21] Mirimanoff RO, Sinzig M, Krüger M, Miralbell R, Thoni A, Ries G, Bosset J-F, Bernier J, Bolla M, Nguyen TD, Lütolf UM, Hünig R, Kurtz J, Greiner R, Coucke PA (1993) Prognosis of human chorionic gonadotropin-producing seminoma treated by postoperative radiotherapy. Int J Radiat Oncol Biol Phys 27: 17–23 8365938 10.1016/0360-3016(93)90416-s

[bib22] Niewald M, Waziri A, Walter K, Nestle U, Schnabel K, Humke U (1995) Low dose radiotherapy for stage I seminoma: early results. Radiother Oncol 37: 164–166 8747942 10.1016/0167-8140(95)01631-p

[bib24] Oliver RTD, Edmonds PM, Ong JYH Ostrowski MJ, Jackson AW, Baille-Johnson H, Williams MV, Wiltshire CR, Mott T, Prat WR, Trask CWL, Hope-Stone HF (1994) Pilot studies of 2 and 1 courses of carboplatin as adjuvant for stage I seminoma: should it be tested in a randomized trial against radiotherapy? Int J Radiat Oncol Biol Phys 29: 3–8 8175442 10.1016/0360-3016(94)90219-4

[bib23] Oliver RTD, Ong J (1996) Seventeen years experience of phase I/II studies of single agent platinum compounds as alternatives to radiation for metastatic and stage I seminoma. Proc Am Soc Clin Oncol 15: 80

[bib25] Read G, Johnston RJ (1993) Short duration radiotherapy in stage I seminoma of the testis: preliminary results of a prospective study. Clin Oncol 5: 364–366 10.1016/s0936-6555(05)80087-1 8305356

[bib26] Sultanem K, Souhami L, Benk V, Bahary JP, Roman T, Shenouda G, Freeman C (1998) Para-aortic irradiation only appears to be adequate treatment for patients with stage I seminoma of the testis. Int J Radiat Oncol Biol Phys 40: 455–459 9457835 10.1016/s0360-3016(97)00733-5

[bib27] Travis LB, Curtis RE, Storm H, Hall P, Holowaty E, Van-Leeuwen FE, Kohler BA, Pukkala E, Lynch CF, Andersson M, Bergfeldt K, Clarke EA, Wiklund T, Stoter G, Gospodarowicz M, Sturgeon J, Fraumeni JF, Boice JD (1997) Risk of second malignant neoplasms among long-term survivors of testicular cancer. J Natl Cancer Inst 89: 1429–1439 9326912 10.1093/jnci/89.19.1429

[bib29] Vallis KA, Howard GC, Duncan W, Cornbleet MA, Kerr GR (1995) Radiotherapy for stages I and II testicular seminoma: results and morbidity in 238 patients. Br J Radiol 68: 400–405 7795977 10.1259/0007-1285-68-808-400

[bib28] Warde R, Gospodarowicz MK, Banerjee D, Panzarella T, Sugar L, Catton CN, Sturgeon JFG, Moore M, Jewett MAS (1997) Prognostic factors for relapse in stage I testicular seminoma treated with surveillance. J Urol 157: 1705–1710 9112510

[bib30] Warde P, Specht L, Horwich A, Oliver T, Panzarella T, Gospodarowicz M, Maase von der H (2002) Prognostic factors for relapse in stage I seminoma managed by surveillance: a pooled analysis. J Clin Oncol 20: 4448–4452 12431967 10.1200/JCO.2002.01.038

[bib31] Willich N, Wendt T, Rohloff R, Feist H, Meyer-Lenschow T, Lissner J (1986) Zur Strahlentherapie des Seminoms: kleinvolumige Bestrahlung im Stadium pT1N0M0-prophylaktische Mediastinalbestrahlung. Strahlenther Onkol 162: 735–741 3810465

[bib32] Zagars GK, Babaian RJ (1987) Stage I testicular seminoma: rationale for postorchiectomy radiation therapy. Int J Rad Oncol Biol Phys 13: 155–162 10.1016/0360-3016(87)90122-2 3818383

